# A SiO_2_ layer on PEO-treated Mg for enhanced corrosion resistance and bone regeneration

**DOI:** 10.3389/fbioe.2022.1053944

**Published:** 2022-12-23

**Authors:** Longhai Qiu, Chi Zhang, Xiaoming Yang, Feng Peng, Yuliang Huang, Yue He

**Affiliations:** ^1^ Department of Traumatology and Orthopaedic Surgery, Institute of Orthopaedics, Huizhou Central People’s Hospital, Huizhou, China; ^2^ The Second School of Clinical Medicine, Southern Medical University, Guangzhou, China; ^3^ Department of Orthopedics, Guangdong Provincial People’s Hospital, Guangdong Academy of Medical Sciences, Guangzhou, China; ^4^ Department of Orthopaedics, The Quanzhou First Hospital Affiliated to Fujian Medical University, Quanzhou, China

**Keywords:** magnesium, plasma electrolytic oxidation, bone regeneration, corrosion resistance, surface modication

## Abstract

Magnesium (Mg) is a promising biodegradable metal for orthopedic applications, and plasma electrolytic oxidation (PEO) has been widely studied as a corrosion protection coating on Mg-based implants. However, the porous structures and easily formed cracks in fluid are disadvantageous for long-term corrosion protection. In this study, a SiO_2_ layer was deposited on PEO-treated Mg to inhibit the formation of cracks on the PEO layer and prevent the permeation of corrosive fluid. The SiO_2_ layer did not alter the surface morphology of the PEO layer but considerably enhanced its corrosion resistance. The *in vitro* culture of MC3T3-E1 cells demonstrated the good cytocompatibility and osteogenic induction ability of SiO_2_-coated PEO-treated Mg, which could be attributed to Mg and Si ions released from the coating. The coating also favored the angiogenesis behaviors of HUVEC. Furthermore, with the continuous release of Mg and Si ions, the as-prepared implant showed a superior osseointegration ability in a rat bone implantation model. In summary, this newly designed Mg-based implant shows promising potential for orthopedic applications.

## 1 Introduction

In recent decades, non-degradable metals such as titanium (Ti) and Ti alloys, cobalt–chromium (Co-Cr) alloys, and tantalum (Ta) have been used very successfully in orthopedic implants (Geetha et al., 2009; Kaur and Singh, 2019; Cakir et al., 2022; Ou et al., 2022). However, after continuous long-term clinical observations, these non-degradable metals presented some inevitable issues. Firstly, the mismatch of the elastic modulus between the non-degradable metals and natural bone (100–250 GPa vs 7–30 GPa) results in stress shielding, inducing bone atrophy (Zhao et al., 2017). Secondly, wearing particles that originate from fluid corrosion or friction between the implant and surrounding tissue could lead to acute inflammation (Toledano-Serrabona et al., 2022). Thirdly, a second surgery to remove the implant after tissue repair would damage the healed tissues and increase the patient’s economic burden (Piattelli et al., 1998). Therefore, with an elastic modulus (40 GPa) similar to natural bone and complete biodegradability in the body, magnesium (Mg) and its alloys can avoid such shortcomings; it is regarded as the next-generation metal for orthopedic applications (Han et al., 2019; Atrens et al., 2020; Wang et al., 2020a).

In 2013, a Mg-based hollow pressurized screw produced by Syntellix AG Company (Germany) was approved by Conformite Europeenne (CE) (Windhagen et al., 2013), and, in 2015, the U&I company (Korean) developed a Mg-based screw for the internal fixation of hand fractures, the product being approved by the Korea Food and Drug Administration (KFDA) (Lee et al., 2016). In 2022, the Magnesium Development Company (MDC) announced that its Mg-based cannulated locking screw for foot and ankle reconstruction had received the Food and Drug Administration (FDA) Breakthrough Device Designation. In China, the most pioneering research was launched by Shanghai Jiao Tong University and Dongguan Yi’an Technology Co., Ltd.; their products were JDBM (Jiao Da Biodegradable Mg) and high-purity Mg, both of which have completed clinical trials and are undergoing product registration (Zhang et al., 2012; Zhang et al., 2018; Wang et al., 2020b). Although great progress has been made in the past decade, the rapid degradation of Mg owing to its high chemical activity restricts its wide range of clinical applications (Chen et al., 2021; Gnedenkov et al., 2022a). Such degradation of Mg implants results in an excessively alkaline environment, as well as the excessive accumulation of Mg ions and hydrogen, which are fatal to bone regeneration. Moreover, rapid degradation damages the mechanical integrity of the implant, resulting in implant failure. Hence, the corrosion protection of Mg-based implants is vital for large-scale clinical applications.

The most convenient and effective strategy for enhancing the corrosion resistance of Mg is surface modification, which includes hydrothermal treatment, plasma electrolytic oxidation (PEO), plasma spray, and fluoride treatment (Gnedenkova et al., 2015; Gnedenkov et al., 2022b). Among these, PEO shows promising potential for clinical application owing to its hardness, strong adhesion to the substrate, favorable wear resistance, and, most importantly, desirable corrosion protection (Yin et al., 2020; Kaseem et al., 2021; Lin et al., 2021). In addition, as an orthopedic implant, its porous structure is favorable for bone ingrowth, thus enhancing the implant's osseointegration. Nevertheless, the porous structures and easily-formed cracks induced by fluid limit the corrosion protection of Mg substrates (Cui et al., 2017; Jiang et al., 2019). For example, Fischerauer et al. (2013) reported that both the untreated and PEO-coated Mg alloy pins degraded over time and vanished completely after being implanted in rat femurs for 12–16 weeks. Therefore, avoiding the side-effects of the porous structure and cracks would be of great significance for clinical applications of PEO coatings on Mg.

Hence, to avoid the cracks formed on PEO-treated Mg when immersed in fluid, as well as to prevent fluid permeating across the PEO porous structure, we deposited a layer of SiO_2_ on PEO-treated Mg. The corrosion behavior and osteogenic induction ability of the double-layered Mg were investigated *in vitro*. In addition, a rat femur implantation was performed to investigate the osseointegration performance *in vivo*.

## 2 Materials and methods

### 2.1 Sample preparation and characterization

Mg plates (Φ = 10 mm and L = 2 mm) were ultrasonically cleaned and then treated using PEO equipment (Pulsetech, China). The electrolyte contained 10 g/L sodium glycerophosphate (C_3_H_7_Na_2_O_6_P) and 12.5 g/L potassium hydroxide (KOH). The constant current, frequency, and duty cycle for the PEO process were set at 0.8A, 1000 Hz, and 10%, respectively. The reaction was terminated at 340 V. Subsequently, the PEO-treated specimens (Mg@PEO) were placed in a Teflon-lined stainless container (Yiyi Machinery Equipment Co., Ltd., Shanghai) with 3 ml tetraethyl orthosilicate (TEOS) and 3 ml ammonia solution. Then, the container was placed in an oven (Jiecheng Experiment Apparatus Co., Ltd., Shanghai) for 24 h at a temperature of 45°C. The obtained specimens were labeled “PEO@SiO.”

Scanning electron microscopy (SEM; S-3400 N, HITACHI, Japan), energy-dispersive spectrometry (EDS; IXRF-550i, IXRF systems, USA), X-ray photoelectron spectroscopy (XPS; RBD upgraded PHI-5000C ESCA system, Perkin-Elmer, USA), and X-ray diffraction (XRD; D2PHASE, Bruker, USA) were used to characterize the samples.

### 2.2 Water contact angle

A drop of deionized water (2 μL) was gently placed on various sample surfaces, and the contact angles were determined using an optical contact angle system (Model SL200A/B/D).

### 2.3 Ion release

The Mg, Mg@PEO, and PEO@SiO sample groups with four parallel specimens were placed in a bottle with deionized water (10 ml) at 37°C. The supernatant was collected at 1, 4, 7, and 10 days, and fresh deionized water was added at each time point. The concentrations of Mg and Si ions in the supernatant were detected using an Inductively Coupled Plasma Optical Emission Spectrometer (ICP-OES; Thermo Fisher Scientific, USA).

### 2.4 Corrosion resistance evaluation

An electrochemical analyzer (CHI760C, Shanghai, China) with a three-electrode system containing a reference electrode, a counter electrode, and a working electrode was used to evaluate corrosion resistance. The samples with an exposed area of 0.255 cm^2^ were first stabilized in 180 ml of phosphate buffer saline (PBS) to obtain a stable open circuit potential. The potentiodynamic polarization was then measured with a sweep rate of 10 mV/s and a range of −2 V to 0 V. The corrosion current was calculated according to Tafel extrapolation.

In addition, the samples were immersed in PBS (each specimen immersed in 10 ml PBS) at 37°C for various periods and the corrosion morphologies were observed using SEM.

### 2.5 Hemocompatibility assay

Hemocompatibility assays were conducted as previously described (Peng et al., 2016). Fresh blood from healthy New Zealand rabbits was collected and diluted in saline solution (volume ratio of blood/saline was 4:5). Mg, Mg@PEO, and PEO@SiO samples were placed in 1.5 ml of saline solution for 30 min at 37°C. Subsequently, 30 μL of diluted blood was added to each group and incubated for another 60 min. Meanwhile, 30 μL of diluted blood added to either saline or distilled water were used as the negative and positive controls, respectively. The supernatant was thereafter transferred to a new 96-well plate, and the absorbance was measured at 545 nm. The hemolysis rate (HR) was calculated as follows:

$$HR=\left(A_{sample}-A_{negative}\right)/\left(A_{positive}-A_{negative}\right)\times 100\%$$

### 2.6 Cytocompatibility evaluation

The cell culture extracts were prepared by immersing Mg, Mg@PEO, and PEO@SiO samples in cell culture medium at a ratio of 1.25 cm^2^/ml for 24 h at 37°C. The culture extracts were thereafter stored at 4°C until further use. The pre-osteoblast subclone 14 (MC3T3-E1) was cultured in Minimum Essential Medium Alpha (α-MEM; Gibco, Thermo Fisher Scientific) supplemented with 10% fetal bovine serum (FBS; Gibco) at 37°C. MC3T3-E1 cells (1 × 10^4^) were seeded in a 96-well plate for 24 h, after which the culture supernatant was replaced with cell culture extracts. Cell proliferation was evaluated after 1, 3, and 7 days of incubation using the CCK-8 assay (DOJINDO), and the absorbance was measured at 450 nm. The viability of cells cultured with different extracts was detected using a live/dead staining assay. Briefly, MC3T3-E1 cells (1 × 10^4^) were cultured with sample extracts for 1 day; afterwards, live and dead cells were labeled with Calcein-AM (2 μM, green) and propidium iodide (5 μM, red) staining, respectively.

### 2.7 Observation of cytoskeletal organization

The cytoskeletal organization was fluorescently labeled with actin and cell nuclei staining. Briefly, cells in 24-well plates were cultured with different sample extracts for 1 day. After fixing, permeabilization, and blocking, actin and nuclei were stained with phalloidin-rhodamine (KeyGEN BioTech) and DAPI, respectively. Finally, the cytoskeletal organization was imaged.

### 2.8 Alkaline phosphatase (ALP) activity and extracellular matrix mineralization (ECM) assay

MC3T3-E1 cells were seeded in a 24-well plate at a density of 2 × 10^4^ cells/well for complete attachment and then cultured with different extracts supplemented with osteogenic revulsants (ascorbic acid, *ß*-glycerophosphate, and dexamethasone) for osteogenic differentiation. At Days 7 and 14, intracellular ALP was detected using a BICP/NBT ALP staining kit (Beyotime Biotechnology), and ALP activity was qualitatively determined by the insoluble formazan color. On the other hand, the cells were fixed with 75% ethanol after culture with different extracts, and then stained with Alizarin Red S (ARS, 40 nM). Subsequently, images of the red calcium nodules were photographed.

### 2.9 Real-time polymerase chain reaction (RT-PCR)

Pre-osteoblasts were osteogenically induced with the different extracts for 7 and 14 days, as described above. The total RNA of the adherent cells was isolated, and 500 ng of RNA was reverse transcribed using the PrimeScript™ reagent kit (Takara). The mRNA expression levels of osteogenesis-related genes were detected using SYBR Green qPCR Master Mix (Trans, China). The genes included Runt-related transcription factor 2 (Runx2), ALP, Osteocalcin (OCN), and Collagen I (COL I). The primer sequences are listed in Supplementary Table S1.

### 2.10 Angiogenesis differentiation

Human umbilical vein endothelial cells (HUVECs) were cultured in *a*-MEM containing 10% FBS in a humidified, 5% CO_2_ incubator at 37°C. For the cell migration assay, HUVECs were starved overnight with a serum-free culture medium, and then 2 × 10^5^ cells/well were seeded into a six-well plate for attachment. Afterwards, pipette tips were used to make an artificial scratch at the center of the cellular monolayer. The migration of cells was photographed at 0 and 24 h after the cell culture medium was replaced with various extracts. The migration rate (%) was calculated as follows: migration ratio (%) = [(original distance—remaining distance)/original distance] × 100%. For the transwell migration assay, 2 × 10^4^ cells/well were seeded in the upper chamber with 8 μm pore filters (Corning), and different sample extracts were added to the lower chamber as the chemoattractant for 24 h. The cells that migrated to the opposite side of the upper chamber were fixed and stained with 0.2% crystal violet, and the staining cells were imaged for analysis. For the RT-PCR assay, total RNA was isolated from the adherent HUVECs treated with different sample extracts. The mRNA expression levels of vascular endothelial growth factor (*VEGF*) and hypoxia-inducible factor *a* (*HIF-α*) (angiogenesis-related genes) were analyzed using RT-PCR.

### 2.11 Osseointegration evaluation

Eighteen Sprague-Dawley (SD) rats were randomly assigned to the Mg, Mg@PEO, and PEO@SiO groups. The implanted materials were 2 mm in diameter and 8 mm in length. After anesthetization with 3% pentobarbital sodium and sterilization with 2% iodine before the operation, holes were drilled along the medial medullary cavity of the femoral condyle for implantation. The wounds were then sutured and gently disinfected, and the femurs with implants were harvested and fixed at 3 months after surgery. The femur layers around the implants were continuously scanned and reconstructed using micro-CT (Inveon™ Multimodality), and the bone volume/tissue volume (BV/TV) and number of trabeculae (TbN) were also calculated. The collected femurs were then embedded with polyethyl methacrylate, sliced, and subjected to Van Gieson’s (VG) staining. Alternatively, the fixed femurs with implants were decalcified, and the specimens were subjected to immunohistochemical staining of OCN, OPN, and VEGF proteins (1:500, Bioss, China).

### 2.12 Statistical analysis

All *in vitro* studies were repeated at least three times. Data are presented as the mean ± standard deviation. Differences among groups were analyzed by one-way and two-way ANOVA followed by Tukey’s *post hoc* test using SPSS 19.0 software. *p* < 0.05 was considered a significant difference.

## 3 Results and discussion

### 3.1 Surface characterization and corrosion resistance of the PEO@SiO coating

**FIGURE 1:**
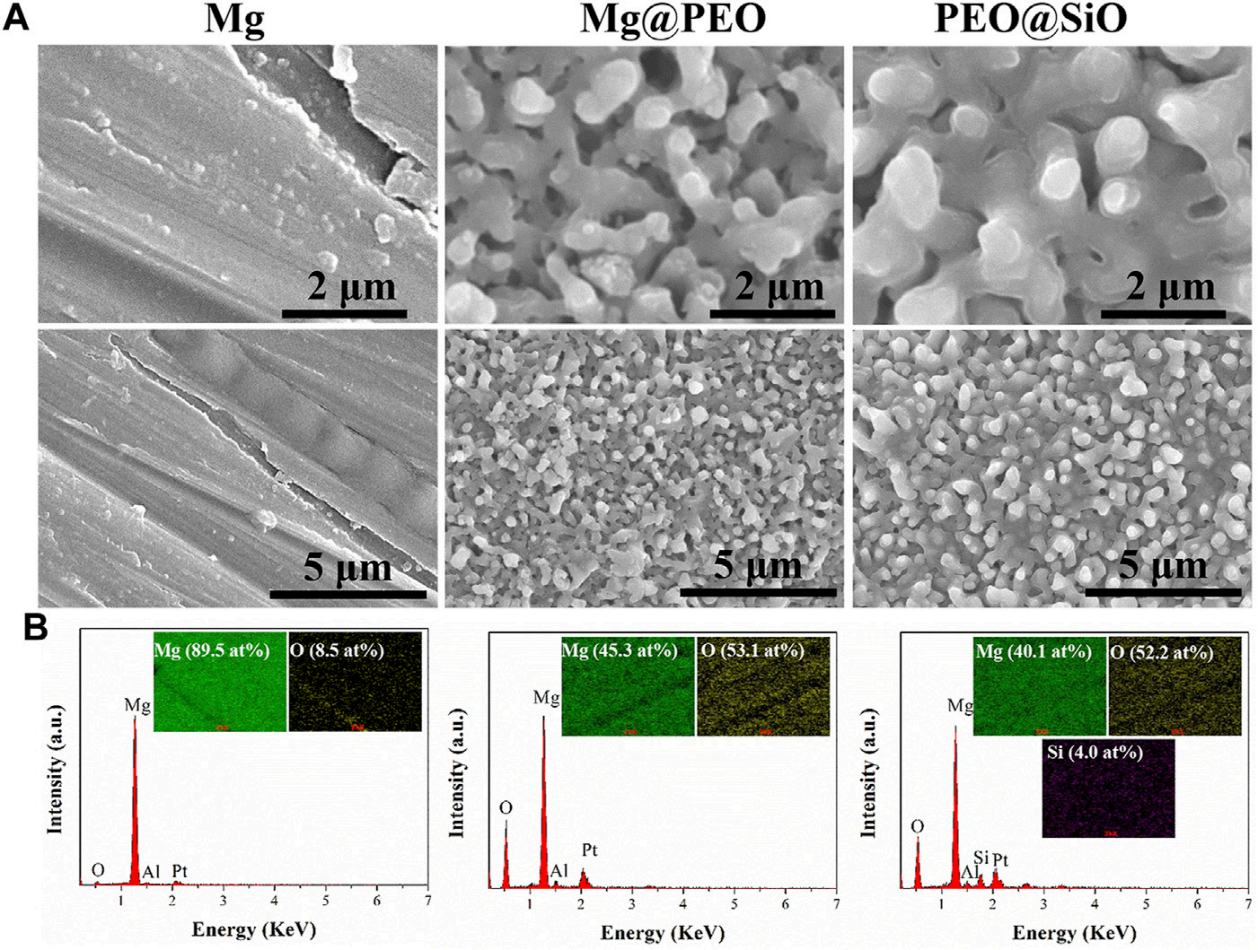
**(A)** Surface morphologies and **(B)** element compositions of the Mg, Mg@PEO, and PEO@SiO samples.

**FIGURE 2:**
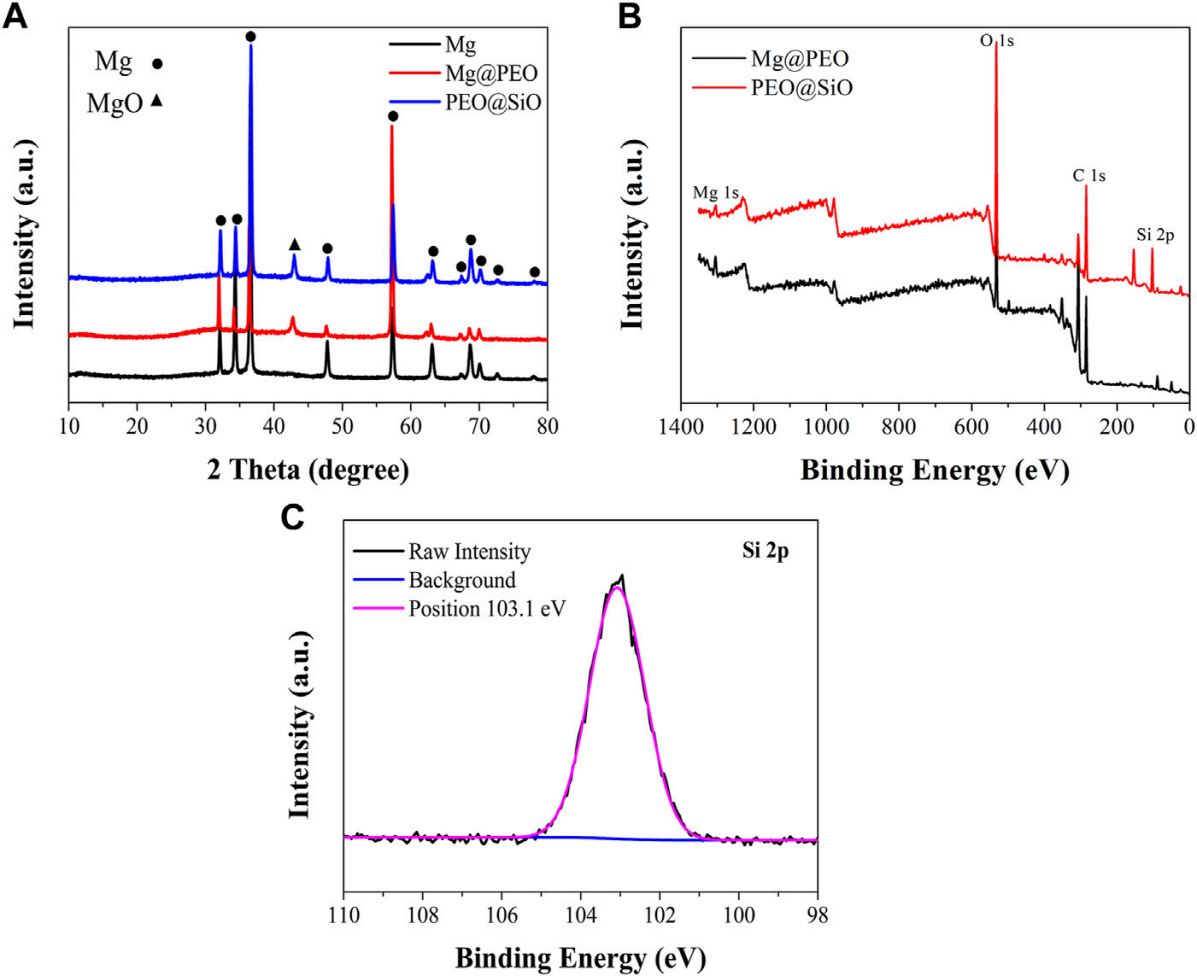
**(A)** XRD patterns of the Mg, Mg@PEO, and PEO@SiO samples. **(B)** Full XPS spectra of the Mg@PEO and PEO@SiO samples. **(C)** High-resolution spectrum of Si 2p for the PEO@SiO sample.

The surface views of various samples are presented in Figure 1A. Similar porous structures were observed on the Mg@PEO and PEO@SiO surfaces. The elemental composition and distribution of the samples are shown in Figure 1B. Only O and Mg were detected in the Mg and Mg@PEO samples. The O element of the Mg sample originated from the natural oxidation of the Mg substrate, whereas the O in the Mg@SiO sample can be attributed to the PEO layer. For the PEO@SiO sample, approximately 4 at% Si was evenly distributed on the surface, indicating successful deposition of the SiO_2_ layer. Weak Al peaks were observed for all the samples, originating from the Mg substrate. Moreover, the detection of Pt was due to the spraying process of Pt before the SEM observation. Figure 2A displays the XRD patterns of the different groups. All the feature peaks in the Mg spectrum are attributed to Mg. Mg@PEO and PEO@SiO exhibited similar characteristic peaks, and both the Mg and MgO phases were detected. No Si-related phases were detected because the SiO_2_ layer was deposited too thinly to be detected. The full XPS profiles are shown in Figure 2B. The Si element only appeared in the PEO@SiO spectrum, which was consistent with the EDS results (Figure 1B). The high-resolution Si 2p spectrum was centered at 103.1 eV (Figure 2C), representing the Si-O band. These results confirmed the formation of the SiO_2_ layer on the PEO-treated Mg surface. Both TEOS and ammonia are volatile substances, especially in an atmosphere of 45°C. Near the PEO surface, ammonia catalyzes TEOS to generate SiO_2_ according to the formula (C_2_H_5_O)_4_Si+2H_2_O→4C_2_H_5_OH + SiO_2_. The generated SiO_2_ is then deposited on the PEO surface.

**FIGURE 3:**
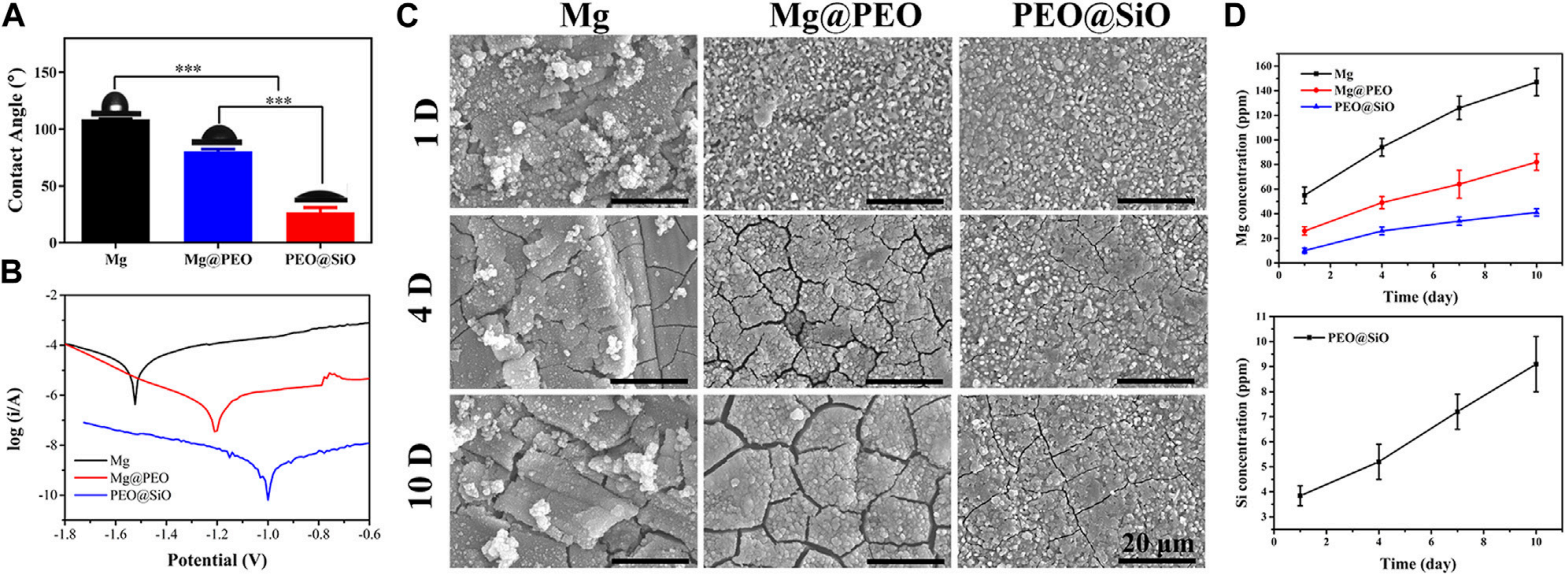
**(A)** Water contact angle, **(B)** potentiodynamic polarization curves, and **(C)** corrosion morphology of the Mg, Mg@PEO, and PEO@SiO samples. **(D)** Mg and Si ions released from various samples.

Figure 3A shows the water contact angles of various samples. The Mg, Mg@PEO, and PEO@SiO samples showed that the water contact angles decreased gradually. The main component of PEO coating is MgO, which can easily react with H_2_O to form Mg(OH)_2_. This is a hydrophilic substance, so the samples showed a decreased water contact angle after the PEO treatment. Notably, the water contact angle of the PEO@SiO sample was approximately 25°. The contact angles of various samples showed similar trends when using PBS and 0.9 at% NaCl as fluids (Supplementary Figure S1). A relatively hydrophilic surface is favorable for cell adhesion. Electrochemical analysis and immersion tests were used to investigate the corrosion behavior of the PEO@SiO sample. As shown in Figure 3B, the potentiodynamic polarization curves revealed that the PEO@SiO sample possessed the lowest corrosion current (7.9 × 10^−10^ A), which was three and four orders lower than that of the Mg@PEO (1.8 × 10^−7^ A) and Mg (8.7 × 10^−6^ A) samples, respectively. Furthermore, the corrosion rates of the Mg, Mg@PEO, and PEO@SiO groups were 1.3, 2.6 × 10^−2^, and 1.2 × 10^−4^ mm/year, respectively. Many researchers have focused on the preparation of multi films on PEO-treated Mg to improve its corrosion resistance. For example, Zhang *et al.* (2022) reported a LDH-sealed PEO-treated Mg, whose corrosion current was approximate 1 × 10^−8^ A. Although the SiO_2_ layer in this work is thinner than the LDH layer (judging from the surface morphology), it showed superior corrosion protection. SiO_2_ is an insulating substance, which can significantly inhibit the transfer of electrons between the corrosive fluid and Mg substrate; thus, depositing a SiO_2_ layer on PEO-treated Mg can greatly improve the corrosion protection of Mg substrate. The corrosion rate of silk-coated anodic oxidation-treated Mg was reported to be 0.017 mm/year, which was similar to the value of PEO-coated Mg in this study (Rahman et al., 2021). When the PEO-treated sample was modified with an outer film, the corrosion rate decreased further. As reported by Yang et al. (2022), the corrosion rate of PEO-coated Al (2.781 × 10^−1^ mm/year) decreased four orders after 1H, 1H, 2H, 2H-perfluorodecyltriethoxysilane modified (4.916 × 10^−5^ mm/year). In this study, the corrosion rate of PEO-treated Mg (2.6 × 10^−2^ mm/year) decreased two orders after modification with SiO_2_ (1.2 × 10^−4^ mm/year). The PEO@SiO sample showing an extremely low corrosion rate can be ascribed to two reasons. Firstly, the electrochemical test only took about 5 min, during which the integrity of the insulating SiO_2_ layer was not damaged. Secondly, the outer SiO_2_ layer is an insulating substance, which can significantly inhibit the transfer of electrons between the corrosive fluid and Mg substrate. The corroded surfaces of different samples are shown in Figure 3C. A rough corrosion surface and many corrosion products appeared on the Mg surface after 1 day of immersion. When the immersion time was extended to 4 days, many corrosion cracks appeared on the Mg@PEO surface; after 7 days, wider corrosion cracks were observed on the Mg@PEO surface. However, after depositing a SiO_2_ layer on the PEO surface, fewer cracks were generated after 4 days, and the cracks were also significantly inhibited after 7 days, compared to the Mg@PEO sample. Obvious corrosion gas was released from the Mg and Mg@PEO samples, whereas no gas was observed from the PEO@SiO surface (Supplementary Figure S2). All these data suggest that the corrosion resistance of PEO@SiO is greatly improved, which can be attributed to the uniformly distributed SiO_2_ layer inhibiting fluid permeation and blocking electro conduction during the corrosion process, as well as inhibiting the formation of corrosion cracks. We further evaluated the Mg and Si ions released from the samples (Figure 3D). The released Mg ions exhibited the following trend: Mg > Mg@PEO > PEO@SiO. Because of the existence of the SiO_2_ layer, the PEO@SiO sample also showed a sustained release of Si ions.

### 3.2 *In vitro* performance of the PEO@SiO coating

**FIGURE 4:**
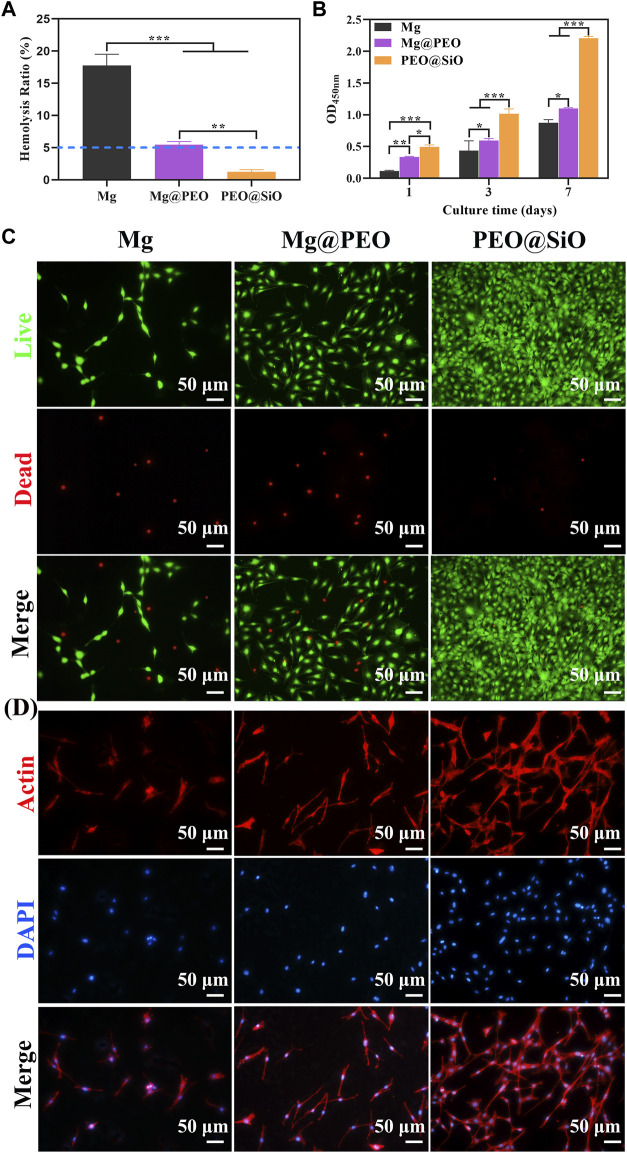
*In vitro* evaluation of biocompatibility: **(A)** Hemolysis rate (%) of Mg, Mg@PEO, and PEO@SiO samples. **(B)** Cell proliferation evaluated by CCK-8 assay for 1, 3, and 7 days. **(C)** Fluoroscopy images of live/dead (green/red) staining of MC3T3-E1 cultured on various samples for 1 day. **(D)** Fluoroscopy images of actin cytoskeleton stained with phalloidine (red) and the nucleus stained with DAPI (blue) of MC3T3-E1 cells cultured on various samples for 1 day.

This study primarily considered the biocompatibility, including the hemo and cytocompatibility, of the surface coatings on Mg. Hemolysis tests can evaluate the effects of blood-contacting implants or devices on blood components; the HR is expected to be less than 5% for clinical application according to the DINISO 10993-4 standard (Nalezinková, 2020). Figure 4A shows the HR values of different samples. The HR (<5%) of SiO_2_-layer modified Mg was significantly improved compared with Mg and PEO-coated Mg (*p* < 0.01**)**, which met the criteria for hemocompatibility.

The behavior of bone-related cells is regulated by the microenvironment constructed by ion release and pH changes from Mg implantation. Therefore, the cytocompatibility of Mg implants is closely related to its corrosion properties. The pH values and ion concentration of the extracts were detected. According to the ICP-AES results (Supplementary Figure S3), the Mg-ion concentration of the PEO@SiO group (85.9 ppm) was significantly lower than the PEO-coated samples (181.3 ppm) and bare Mg (118 ppm), indicating the superior corrosion resistance of the PEO@SiO coating. The concentration of Si ions was 29.5 ppm. As shown in Supplementary Figure S4, the pH values of the bare Mg, Mg@PEO, and PEO@SiO groups increased to 8.3, 8.15, and 7.91, respectively, compared with the cell culture medium. The cytocompatibility of the samples was evaluated using a pre-osteoblast cell line. Cell proliferation was assessed after incubation with different sample extracts. Figure 4B shows that cells in the Mg, Mg@PEO, and PEO@SiO groups continued to proliferate for 7 days and that the number of living cells was in the following order: PEO@SiO > Mg@PEO > Mg. It is clear that cells in the PEO@SiO group had a significantly higher proliferation rate than those in the other groups after incubation. To verify cell viability, cells cultured in different extracts for 1 and 7 days were subjected to live/dead fluorescent staining assay. Only a few viable cells adhered to the Mg group, and the number of viable cells (green fluorescence) increased after PEO treatment (Figure 4C and Supplementary Figure S5). In contrast, denser and more viable cells were distributed in the PEO@SiO group, which was consistent with the cell proliferation results. The cells directly cultured on the sample’s surface for 1 day showed the same trend (Supplementary Figure S6). Furthermore, cell morphology was observed by cytoskeletal actin staining (Figure 4D). After 24 h of incubation, sporadically visible cells that adhered to the Mg group were still spherical. However, more cells were spread over the Mg@PEO and PEO@SiO groups. In particular, a greater number of lamellipodia and filopodia (red fluorescence) were distributed in the PEO@SiO group.

**FIGURE 5:**
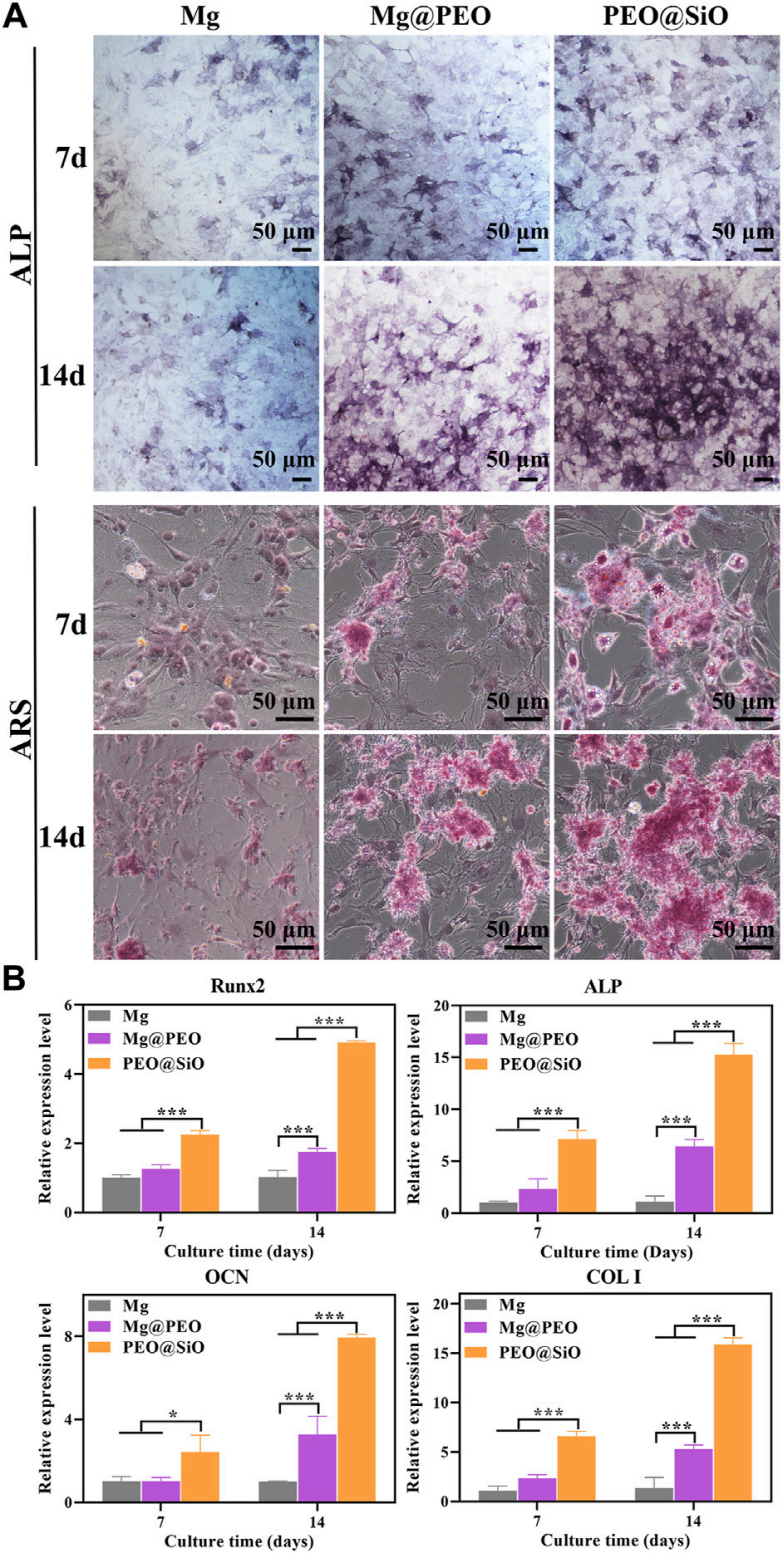
*In vitro* evaluation of osteogenesis: **(A)** Osteogenic differentiation was evaluated using ALP staining, and extracellular matrix mineralization was evaluated using Alizarin Red staining of MC3T3-E1 cultured on various samples for 7 and 14 days, respectively. **(B)** Relative mRNA expression of osteogenesis-related genes including Runx2, ALP, OCN, and COL I for MC3T3-E1 cultured on various samples for 7 and 14 days; ^*^ *p* < 0.05 and ^***^ *p* < 0.001.

The osteogenic responses of the PEO@SiO, Mg@PEO, and Mg groups were examined using classic ALP activity and ECM deposition assays. ALP, as the initial marker of osteogenic differentiation, is produced by osteogenic cells, and bone formation is enhanced with an increase in ALP activity. Consequently, osteogenic differentiation was primarily evaluated by ALP staining. As shown in Figure 5A, ALP activity increased markedly in the Mg@PEO and PEO@SiO groups after 7–14 days of incubation, with the densest and most uniform purple-blue staining of cells treated with PEO@SiO extracts. However, ALP staining was not obvious in the Mg group, indicating the considerable effects of the PEO@SiO coating on early osteogenic differentiation. ECM is another characteristic of osteoblasts and is accompanied by the formation of calcium nodules. Calcium and other inorganic salts deposit into osteoid in the form of hydroxyapatite crystals under certain conditions; normal bone is then gradually formed (Lin et al., 2018). Therefore, calcium deposition in MC3T3-E1 cells was observed using ARS staining after incubation for 7 and 14 days. In line with ALP activity, the densest and most extensive red nodules were observed in the PEO@SiO group. The numbers of colored ECM were in the following order: PEO@SiO > Mg@PEO > Mg (Figure 5A).

In addition to osteogenic phenotypes such as ALP and ECM, osteogenicity is regulated by a series of transcription factors and osteogenesis-related genes at the molecular levels. As a transcription factor, Runx2 is expressed by mesenchymal stem cells, osteoblasts, and chondrocytes in the early stages of wound healing. Binding of Runx2 with osteoblast-related cis-acting elements promotes the expression of OCN, OPN, COL I, and bone sialoprotein. Therefore, Runx2 not only regulates osteogenic differentiation but also participates in osteoblast functions (Gomathi et al., 2020). As the main non-collagenous protein in bone tissue, OCN is specifically secreted by osteoblasts, while COL I is the most important collagen fiber component in the bone matrix. Both these factors can fully reflect osteoblast functions. As shown in Figure 5B, the mRNA expression of osteogenesis-related genes in the Mg@PEO and PEO@SiO groups increased from Days 7–14. In addition, pre-osteoblasts treated with PEO@SiO exhibited the highest levels of Runx2, ALP, OCN, and COL I genes at 7 and 14 days, suggesting that the SiO_2_ layer modification significantly promoted the expression of molecules involved in bone reconstruction. The above observations were largely attributed to the synergistic effect of the corrosion products from the Mg substrate and Si ion release. Proper release of Mg ions and a weak alkaline microenvironment are beneficial for osteogenesis differentiation (Wang et al., 2018a; Mao et al., 2022). Si ions have been reported to be crucial for biomineralization and collagen synthesis (Zhou et al., 2021). Moreover, Si is known as a “biological cross-linker” and plays a particularly important role in bridging proteoglycan and collagen (Bunpetch et al., 2019). As early as the 1970s, Carlisle *et al.* (1970) identified Si as a key element involved in tissue mineralization. Si ions are strongly involved in the initial stages of biomineralization, and decreased intake of Si leads to significantly defective bone and cartilage growth (Götz et al., 2019). Taken together, these findings suggest that the PEO@SiO coating improved osteogenesis by promoting cell differentiation and mineralization.

**FIGURE 6:**
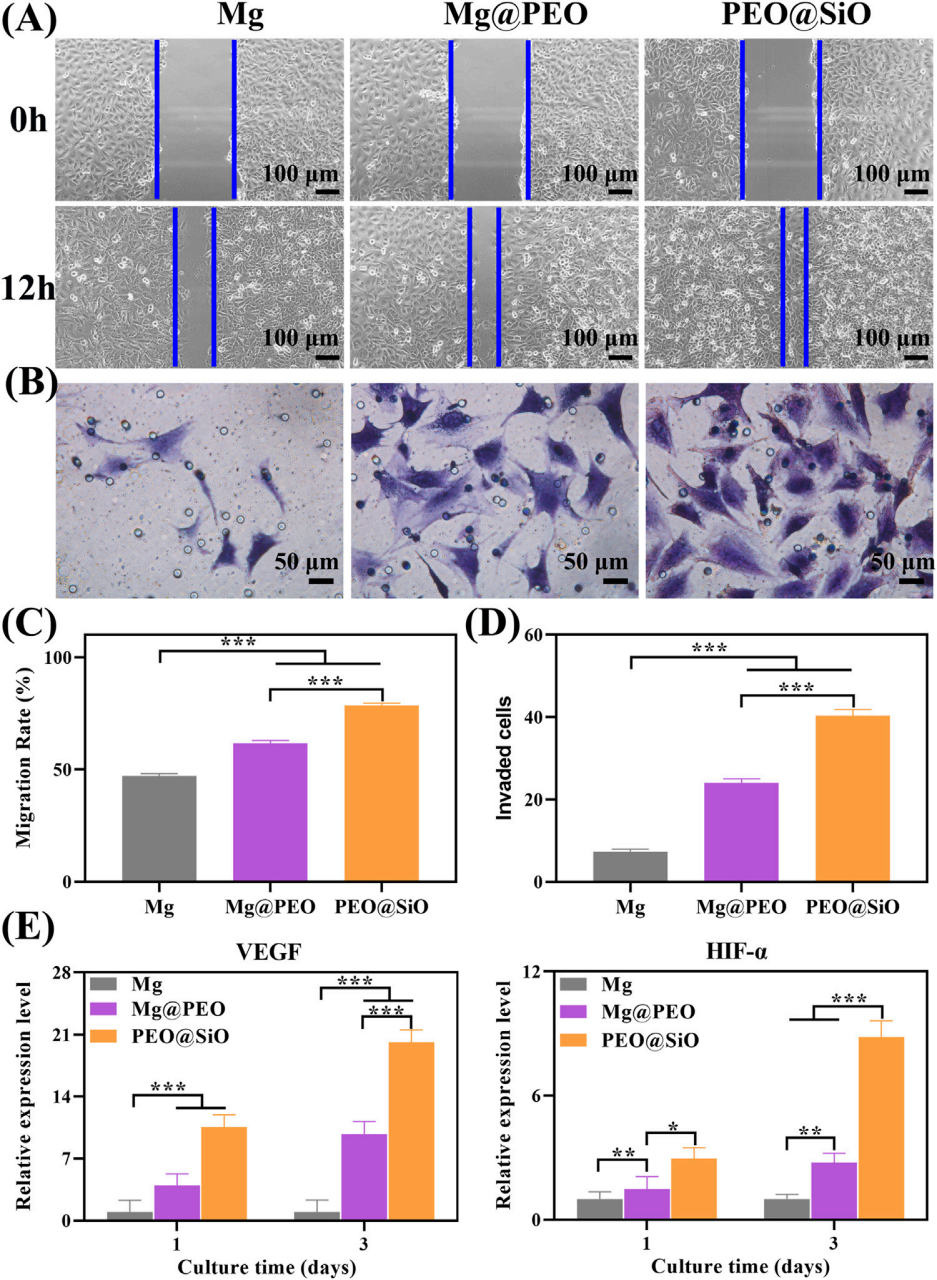
*In vitro* angiogenic ability: **(A)** Migration of HUVECs cultured for 12 h were evaluated by wound healing assay, and corresponding quantitative results are shown in **(C)**; **(B)** Crystal violet staining of migrated HUVECs cultured for 24 h in a transwell assay, with corresponding number of invaded cells is shown in **(D)**; **(E)** Relative mRNA expression of angiogenesis-related genes including VEGF and HIF-α for HUVEC cultured on various samples for 1 and 3 days; ^*^ *p* < 0.05, ^**^ *p* < 0.01, and ^***^ *p* < 0.001.

Blood vessels, especially vascular endothelial cells, control organ growth, balance, regeneration, and neovascularization, which are prerequisites for bone tissue reconstruction and maturation (Kusumbe et al., 2014). Therefore, it is important to evaluate peri-implant vascularization and angiogenic induction by bone implants. As cell migration and invasion are necessary for the angiogenesis of endothelial cells, the effects of our samples on the migration and invasion behavior of HUVECs were assessed using wound healing and transwell assays, respectively. For cell migration, the wounds of the Mg, Mg@PEO, and PEO@SiO groups had all narrowed after 12 h of incubation compared with the original scratches (Figure 6A). Moreover, the gap in the HUVEC scratch in the PEO@SiO group was the smallest among the groups, suggesting the fastest migration ratio. Further quantitative results of the migration rate showed that the migration ratio of the PEO@SiO group was 1.3 and 1.7 times higher than that of the Mg@PEO and Mg groups, respectively (Figure 6C). In addition, the PEO@SiO group promoted cell invasion (Figure 6B), and the number of invaded cells in the PEO@SiO group was 1.7 and 5.5 times higher than that in the Mg@PEO and Mg groups, respectively (Figure 6D). To further investigate the angiogenic effects of the different extracts at the molecular level, the expression of angiogenesis-related genes (*VEGF* and *HIF-α*) was evaluated after 1 and 3 days of incubation. HIF-α signaling is one of the main pathways for blood vessel formation and participates in the entire process of angiogenesis by regulating the expression of downstream growth factors, including nitric oxide synthase, VEGF, and VEGF receptor-1 (Choudhry and Harris, 2018). VEGF can further induce migration and tube formation, thereby promoting neovascularization and increasing vascular permeability (Melincovici et al., 2018). Figure 6E shows that the PEO@SiO group had significantly upregulated expression levels of angiogenesis-related genes. Overall, these findings indicated that the PEO@SiO coating effectively promoted angiogenesis attributed to the release of Si ions. It is well established that Si has a positive effect on angiogenesis *in vitro* and *in vivo* (Mao et al., 2017; Wan et al., 2022). For example, Si doping of biomaterials is beneficial for the proliferation of HUVEC and upregulates the expression of angiogenesis-related genes (Wang et al., 2018b). It is also confirmed that Si ions could stimulate vascularization in the HUVEC-MSC or HUVEC-fibroblast co-culture systems (Wei et al., 2014).

### 3.3 *In vivo* performance of the PEO@SiO coating

**FIGURE 7:**
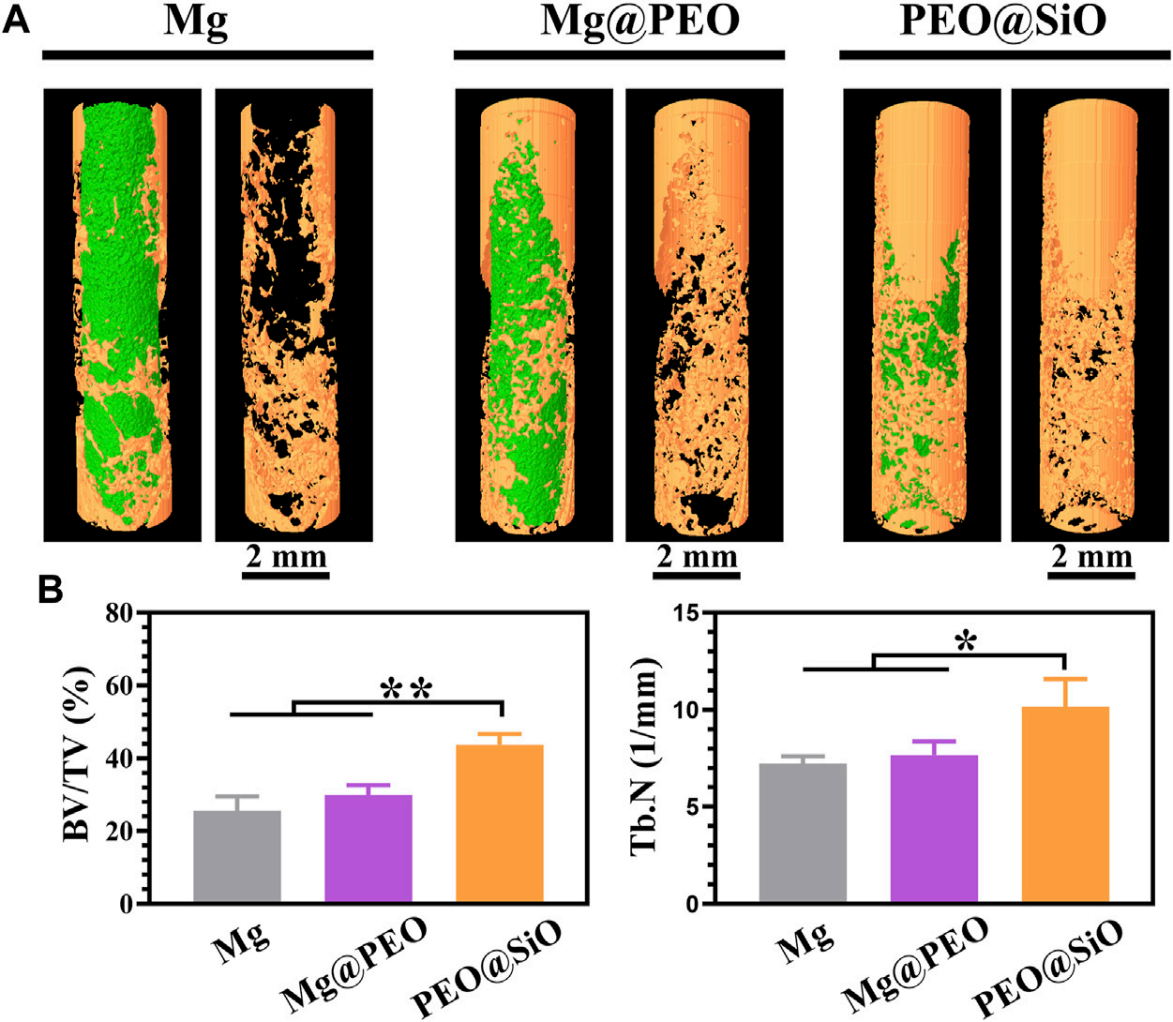
Micro-CT analysis of bone femur obtained after implantation with different samples for 3 months: **(A)** 3D-reconstruction images of the implants and **(B)** corresponding quantitative analysis of the micro-CT data of BV/TV and Tb.N (*p < 0.05; **p < 0.01).

The modulatory role of the PEO@SiO coating in osteogenesis and angiogenesis was confirmed by the above *in vitro* experiment. To further investigate the effect of the PEO@SiO coating on osseointegration *in vivo* around the implants, a femoral implantation model was established, and the implants were harvested after 3 months. It is apparent from the 3D-reconstructed micro-CT images (Figure 7A) that more new bone tissue (indicated by yellow) was observed for the Mg@PEO compared to those observed for Mg implants, and the largest area of new bone tissue was detected for the PEO@SiO implant. The corresponding BV/TV and Tb.N values were also calculated. BV/TV represents the ratio of bone volume to total tissue volume, which can directly reflect the change of bone mass. On the other hand, the value of Tb.N can be calculated from the microstructure of bone trabeculae by using micro-CT reconstruction, and Tb.N significantly increases in the process of bone anabolism. As shown in Figure 7B, both the BV/TV and Tb.N values for the Mg@PEO group were slightly higher than those of the Mg group, but no statistically significant difference was observed. However, the BV/TV and Tb.N values were significantly higher in the PEO@SiO group than those in the other two groups, suggesting its superior osteogenic capability.

**FIGURE 8:**
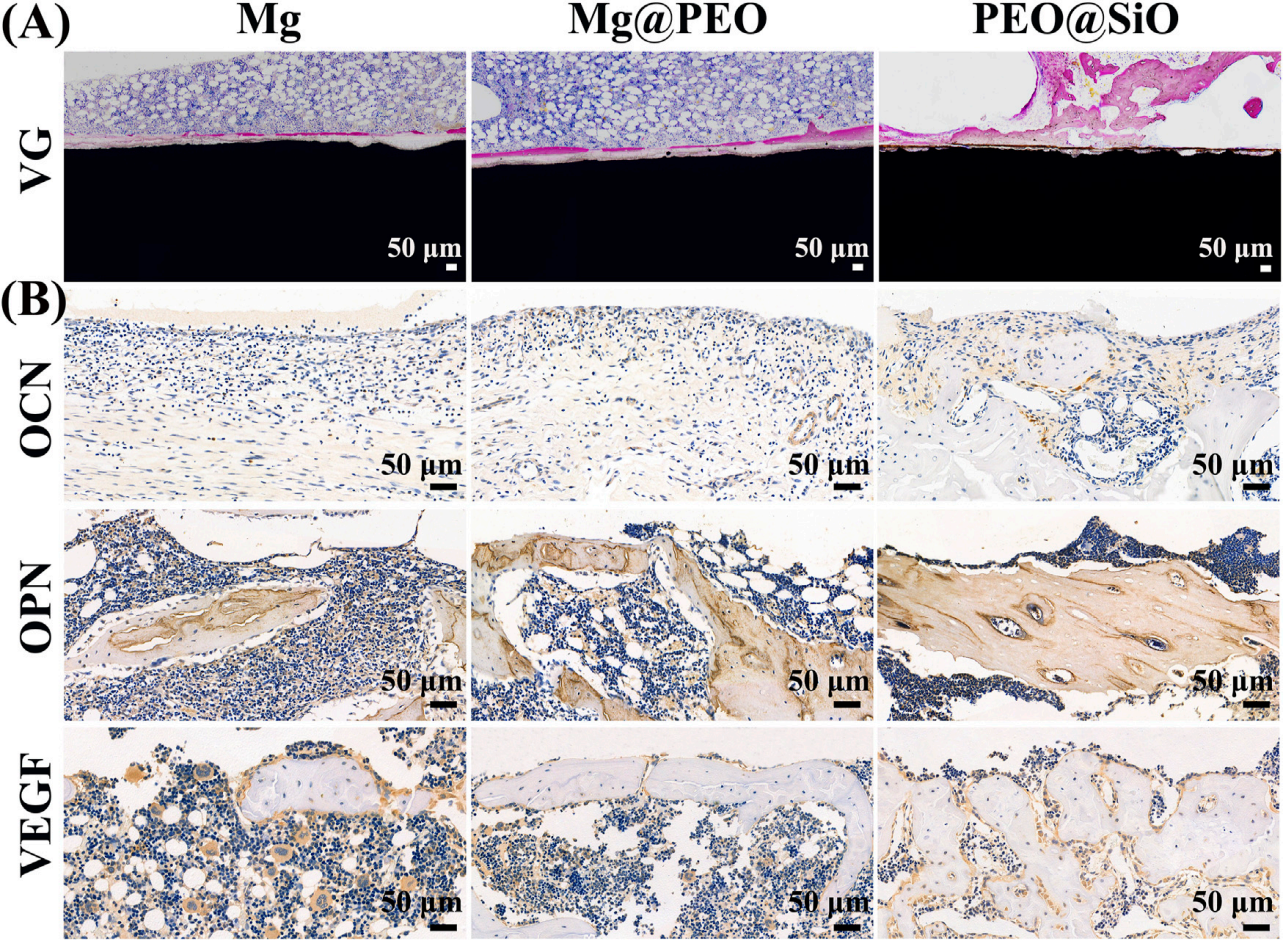
Osseointegration ability of different implants after implantation for 3 months: **(A)** VG staining of tissues surrounding the various samples after femur implantation. **(B)** Immuno-histological staining of OCN, OPN, and VEGF protein of decalcified femurs around different implants.

For histopathological observation, classic VG staining of undecalcified bone sections with implants was performed to visualize osseointegration ability around the interface between the bone and implants. As shown in Figure 8A, very thin and few neotissue layers were distributed around the Mg implants, whereas the thickness of the new bone layer increased around the surface-modified implants. The new bone quantity in the three groups showed the following trend: PEO@SiO > Mg@PEO > Mg. Significantly thicker and more continuous new bones were observed in the PEO@SiO group. Moreover, new bones were more tightly integrated onto the surface of the PEO@SiO implants. Owing to the poor corrosion resistance of the bare Mg implant, the higher ion concentration and pH around the bare Mg implants had adverse effects on osteogenic differentiation. However, the corrosion resistance was significantly improved after PEO modification and SiO_2_ deposition. The release of a small amount of Mg ions and the construction of a weak alkaline microenvironment provides favorable conditions for peri-implant osseointegration (Zhang et al., 2016). As an essential trace element in bone construction, Si ions can induce collagen deposition, apatite synthesis, and angiogenesis *in vivo.* This suggests the synergistic effects of Mg and Si on the *in vivo* osseointegration of the PEO@SiO-modified implant.

Immunohistochemical staining was performed on the decalcified sections to evaluate the expression of osteogenesis and angiogenesis-related proteins (OPN, OCN, and VEGF) around the implants. OCN and OPN are specific markers of osteoid, and more positive staining areas and darker staining color surrounded the PEO@SiO implant compared to the other two implants (Figure 8B), indicating greater OCN and OPN expression induced by the PEO@SiO-coated Mg implant. VEGF is a crucial contributor to the formation of new blood vessels, and a higher VEGF expression was observed in the PEO@SiO group than in the Mg@PEO and Mg groups, suggesting better vascularization around the PEO@SiO implant. Taken together, the expression trends of OCN, OPN, and VEGF followed the order of PEO@SiO > PEO > Mg.

## 4 Conclusion

In this work, a SiO_2_ layer was successfully deposited on PEO-treated Mg. The SiO_2_ layer had few influences on the porous surface morphology of the PEO coating. Nevertheless, the corrosion current is reduced by three orders of magnitude after SiO_2_ was deposited, suggesting that the existence of the SiO_2_ layer significantly enhanced the corrosion resistance of the PEO-treated Mg. Moreover, only a few cracks were observed on the SiO_2_-coated PEO-treated Mg after immersion in PBS for 14 days. With the controlled release of Mg and Si ions, the SiO_2_ modified PEO-treated Mg showed improved osteogenic differentiation capability. Moreover, SiO_2_-coated PEO-treated Mg exhibited superior osteogenesis and osseointegration when implanted in rat femurs. With enhanced corrosion resistance and bone rebuilding ability, the SiO_2_-coated PEO-treated Mg shows promising potential for bone defect therapy.

## Data Availability

The original contributions presented in the study are included in the article/Supplementary Material, further inquiries can be directed to the corresponding authors.
