# Supplementary material for: A SiO2 layer on PEO-treated Mg for enhanced corrosion resistance and bone regeneration
Source: Front Bioeng Biotechnol. 2022 Dec 23;10:1053944. doi: 10.3389/fbioe.2022.1053944 (PMC9816664; doi:10.3389/fbioe.2022.1053944)
Supplement: Supplementary file 1 [file Table1.DOCX]

A SiO_2_ layer on PEO-treated Mg for enhanced corrosion resistance and bone regeneration

Longhai Qiu^a,b^, Chi Zhang^c^, Xiaoming Yang^d^, Feng Peng^c^, Yuliang Huang^a,^*, Yue He^c,^*

*^a^Department of Traumatology and Orthopaedic Surgery, Institute of Orthopaedics,* *Huizhou Central People's Hospital, Huizhou, Guangdong 516001, China.*

*^b^The Second School of Clinical Medicine, Southern Medical University, Guangzhou 510515, China.*

*^c^Department of Orthopedics,* *Guangdong Provincial People's Hospital, Guangdong Academy of Medical Sciences, Guangzhou 510080, China*

*^d^Department of Orthopaedics,* *The Quanzhou First Hospital Affiliated to Fujian Medical University, Quanzhou 362000, China*

**^*^ Corresponding Authors:**

E-mail addresses: 18616936841@163.com (Y.L. Huang); [raul86@126.com](mailto:raul86@126.com) (Y. He)

**Table S1.** Primer sequences used for real-time PCR amplification.

| **Gene name** | **Forward sequence** | **Reverse sequence** |  |
| --- | --- | --- | --- |
| GAPDH (mouse) | TTCCAGGAGCGAGACCCCACTA | GGGCGGAGATGATGACCCTTTT |  |
| Runx2  ALP  OCN  COL I  GAPDH (human)  HIF-α  VEGF | GACTGTGGTTACCGTCATGGC  TCCGTGGGCATTGTGACTAC  GGTAGTGAACAGACTCCGGC  GCTCCTCTTAGGGGCCACT  CAAGAGCACAAGAGGAAGAGAG  TCTACCAGTTGCAGCCTGAC  CAGGACATTGCTGTGCTTTG | ACTTGGTTTTTCATAACAGCGGA  TGGTGGCATCTCGTTATCCG  GGCGGTCTTCAAGCCATACT  ATTGGGGACCCTTAGGCCAT  CTACATGGCAACTGTGAGGAG  GTTCCCTTCCTCCTTGATTT  CTCAGAAGCAGGTGAGAGTAAG | |


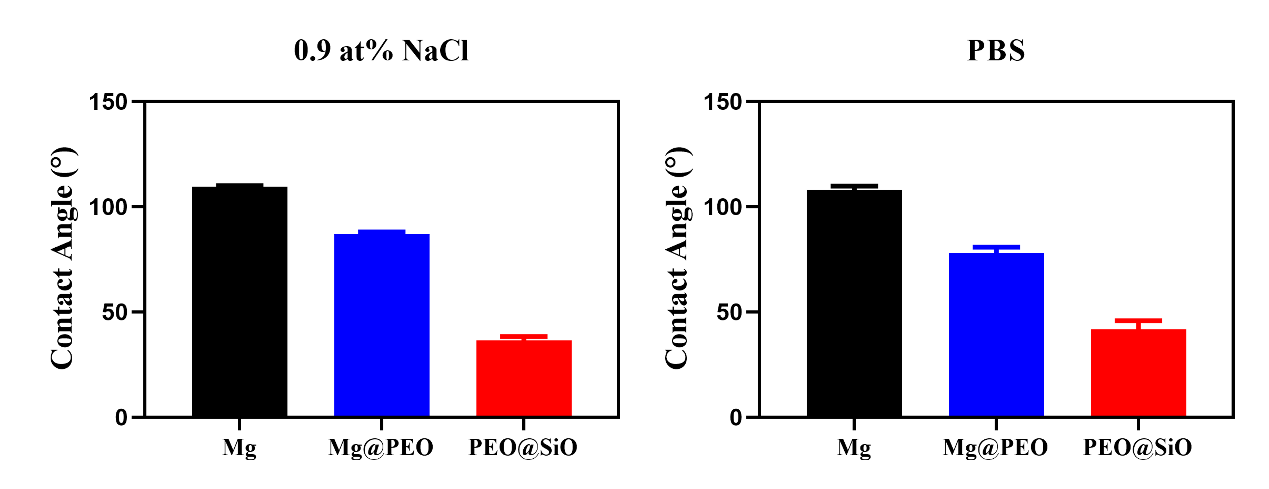


**Figure S1.** The contact angles of the Mg, Mg@PEO, and PEO@SiO samples using PBS and 0.9 at% NaCl as the fluids.

**
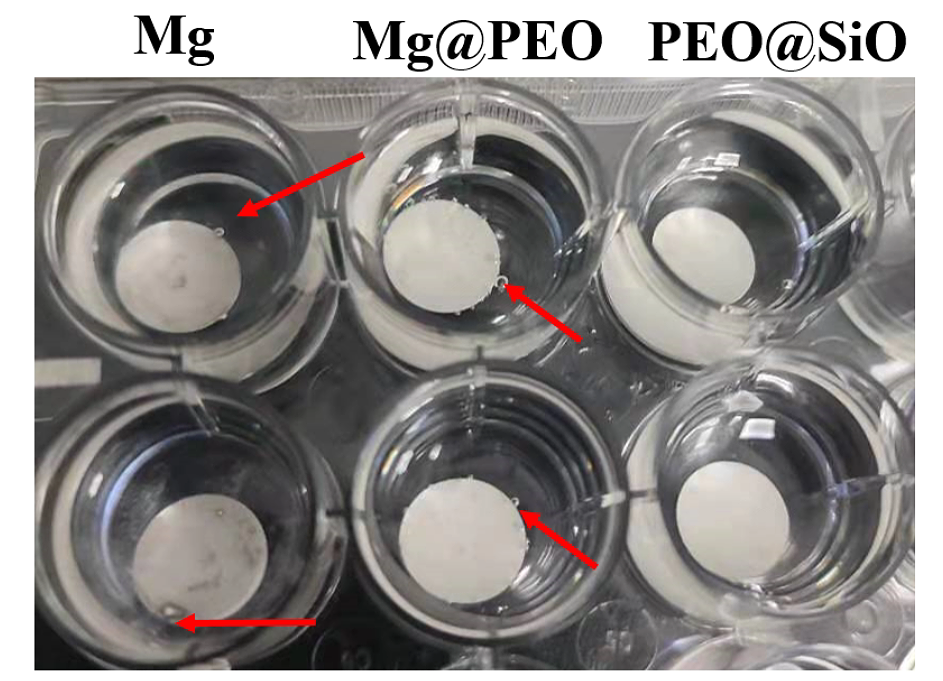
**

**Figure S2.** Optical photos of Mg, Mg@PEO, and PEO@SiO samples after immersed in PBS for 4 days.

**
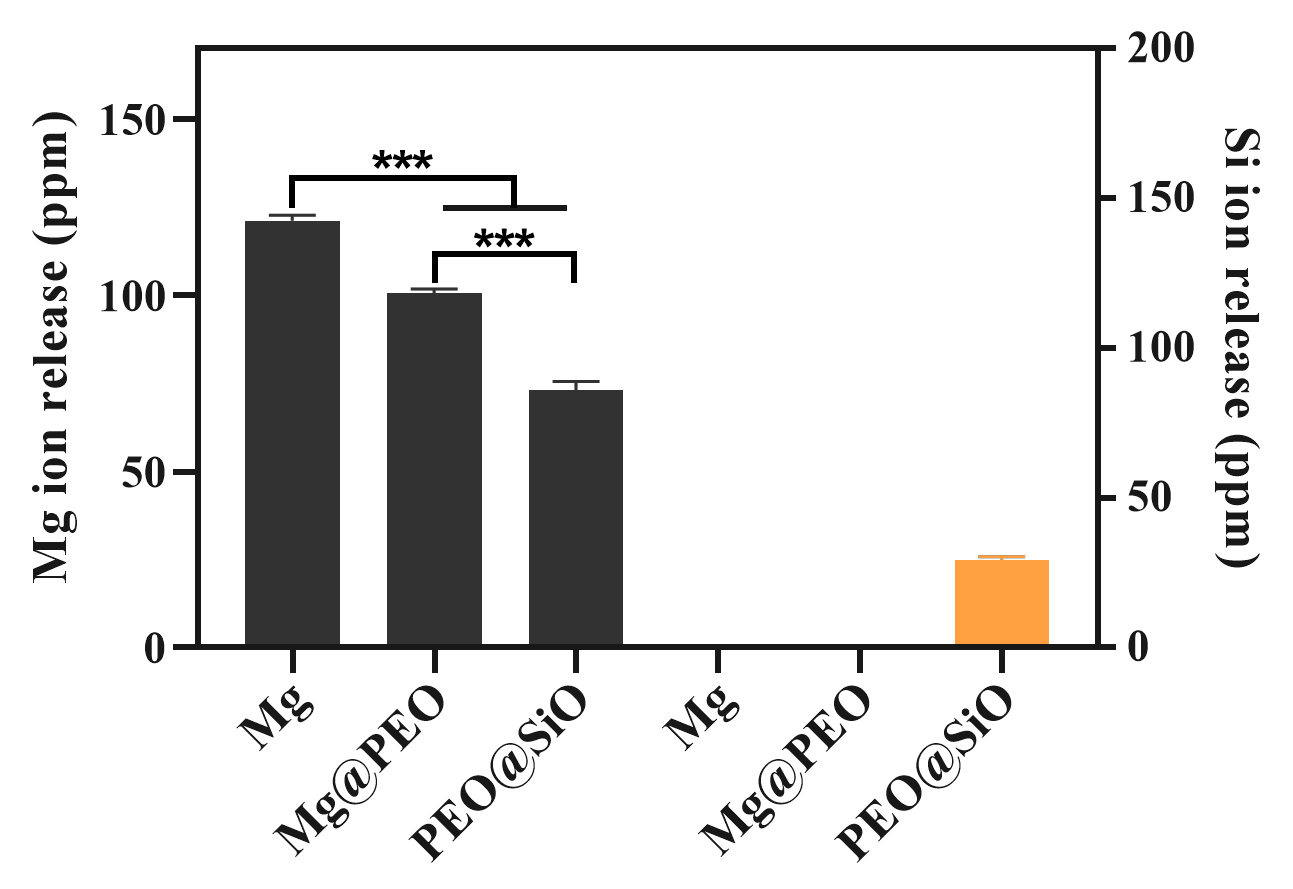
**

**Figure S3**. Mg ions release behavior of Mg, Mg@PEO and PEO@SiO samples immersed in the culture medium for 24 h.

**
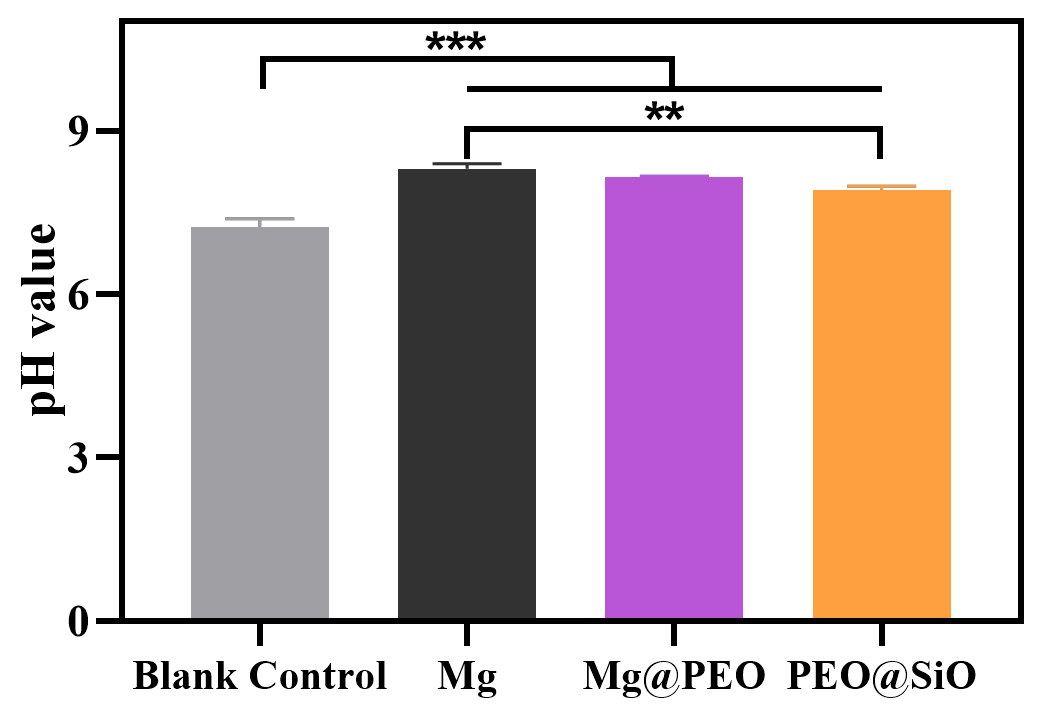
**

**Figure S4**. pH value of the blank control, Mg, Mg@PEO and PEO@SiO groups.

**
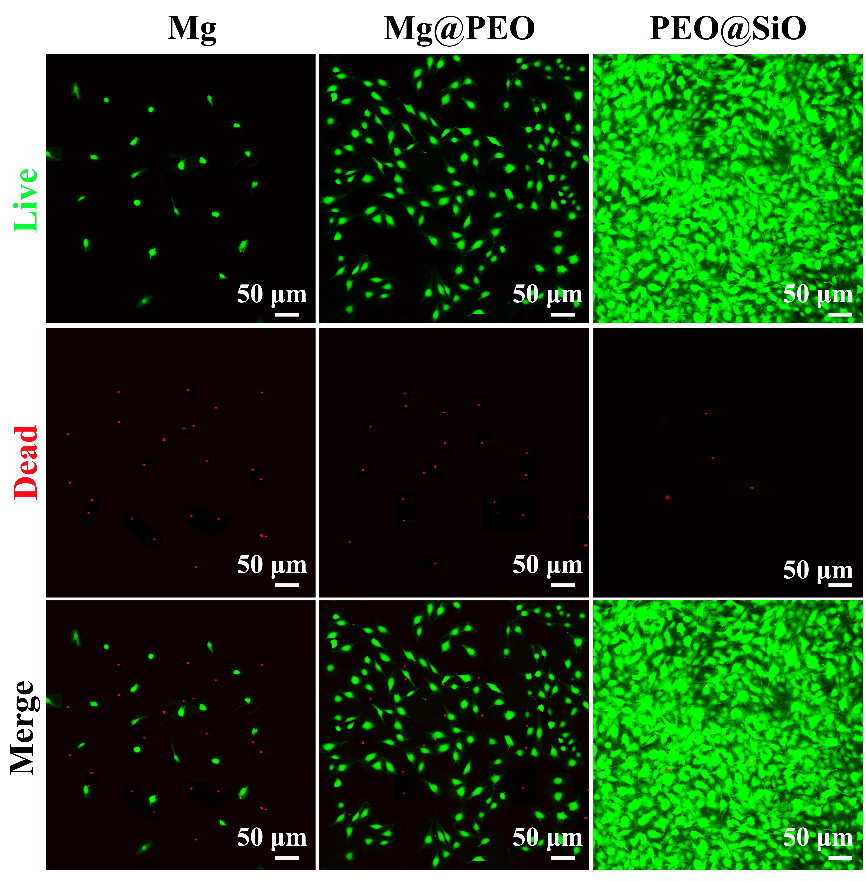
**

**Figure S5.** Fluoroscopy images of live/dead (green/red) staining of MC3T3-E1 cultured with different sample extracts for 7 days.

**
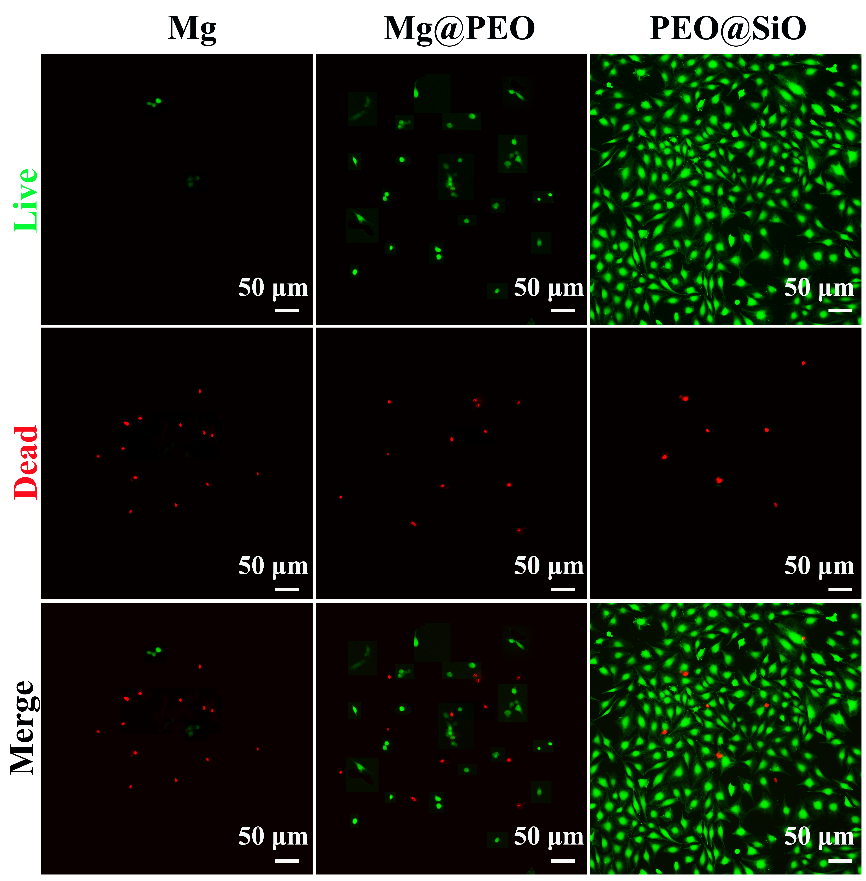
**

**Figure S6.** Fluoroscopy images of live/dead (green/red) staining of MC3T3-E1 cultured on the surface of different samples for 1 day.
